# Hyperglycemia Aggravates Hepatic Ischemia Reperfusion Injury by Inducing Chronic Oxidative Stress and Inflammation

**DOI:** 10.1155/2016/3919627

**Published:** 2016-08-31

**Authors:** Yihan Zhang, Dongdong Yuan, Weifeng Yao, Qianqian Zhu, Yue Liu, Fei Huang, Jiayu Feng, Xi Chen, Yong Huang, Xinjin Chi, Ziqing Hei

**Affiliations:** ^1^Department of Anesthesiology, The Third Affiliated Hospital of Sun Yat-sen University, Guangzhou 510630, China; ^2^Department of Thyroid and Breast Surgery, The Third Affiliated Hospital of Sun Yat-sen University, Guangzhou 510630, China

## Abstract

*Aim*. To investigate whether hyperglycemia will aggravate hepatic ischemia reperfusion injury (HIRI) and the underlying mechanisms.* Methods*. Control and streptozotocin-induced diabetic Sprague-Dawley rats were subjected to partial hepatic ischemia reperfusion. Liver histology, transferase, inflammatory cytokines, and oxidative stress were assessed accordingly. Similarly, BRL-3A hepatocytes were subjected to hypoxia/reoxygenation (H/R) after high (25 mM) or low (5.5 mM) glucose culture. Cell viability, reactive oxygen species (ROS), and activation of nuclear factor-erythroid 2-related factor 2 (Nrf2) and nuclear factor of kappa light polypeptide gene enhancer in B-cells (NF-*κ*B) were determined.* Results*. Compared with control, diabetic rats presented more severe hepatic injury and increased hepatic inflammatory cytokines and oxidative stress. HIRI in diabetic rats could be ameliorated by pretreatment of N-acetyl-L-cysteine (NAC) or apocynin. Excessive ROS generation and consequent Nrf2 and NF-*κ*B translocation were determined after high glucose exposure. NF-*κ*B translocation and its downstream cytokines were further increased in high glucose cultured group after H/R. While proper regulation of Nrf2 to its downstream antioxidases was observed in low glucose cultured group, no further induction of Nrf2 pathway by H/R after high glucose culture was identified.* Conclusion*. Hyperglycemia aggravates HIRI, which might be attributed to chronic oxidative stress and inflammation and potential malfunction of antioxidative system.

## 1. Introduction

Hepatic ischemia reperfusion injury (HIRI) is a major cause for acute postoperative liver dysfunction and liver failure. It is common in major hepatic surgery including liver transplantation and partial hepatectomy. The mechanisms of HIRI consist of multiple complicated pathophysiological processes including mitochondrial energy exhaustion, excessive reactive oxygen species (ROS) production, calcium overload, leukocyte aggregation, cytokine release, and microcirculation dysfunction [1–5]. Of note, the outburst of oxidative and proinflammatory factors is crucial to initiating HIRI [6]. During reperfusion, enormous ROS are generated due to sudden restoration of oxygen and exert direct deleterious effects on cells through lipid peroxidation, protein degradation, and DNA damage [1].

Diabetes is a major risk factor for many surgical complications [7–9]. It is particularly associated with poor prognosis of ischemia reperfusion (IR) injury. Many have reported that IR injury of heart, kidney, and neuron tissues tends to be more severe in diabetic patients [10–12]. Hyperglycemia is a major manifestation of diabetes and it contributes to the progression of many diabetic complications [13].

Hyperglycemia could increase basal ROS levels in cardiomyocytes, renal mesenchymal cells, and endothelial cells, resulting in a state of chronic oxidative stress [14–16]. The increase in baseline ROS generation has been reported to be an important factor to aggravate cardiac IR injury [17]. Furthermore, chronic oxidative stress would diminish the protection of anesthetic preconditioning and ischemic preconditioning upon acute oxidative stress in IR [18, 19].

Chronic oxidative stress may promote the onset or progression of chronic liver diseases including nonalcoholic fatty liver disease (NAFLD), cirrhosis, hepatitis, and hepatic carcinoma, which if not treated properly are likely to advance to end-stage liver diseases requiring surgical intervention [20, 21]. However, little is known about the impact of chronic oxidative stress in diabetes on HIRI. The evidence of correlation between diabetes and negative outcome of HIRI is accumulating [22, 23]. For instance, the risk of liver graft failure is higher in diabetic patients compared with nondiabetic patients after liver transplant [24]. Consistently, diabetes is a risk factor for unexpected readmission in patients receiving hepatic surgery [25]. Since liver plays a central role in glucose metabolism, deterioration of liver function would in turn worsen diabetes [26]. The underlying mechanisms of diabetes and poor outcome of hepatic surgery remain unknown.

As the incidence of diabetes rises rapidly [27], there is a growing need to focus on HIRI in diabetic patients. By establishing in vivo HIRI model on streptozotocin- (STZ-) induced type 1 diabetic rats and in vitro hypoxia/reoxygenation (H/R) model on hepatocytes, we intend to explore the impact of hyperglycemia or high glucose condition upon HIRI or H/R injury and the possible mechanisms.

## 2. Methods

### 2.1. Animal Models of Diabetes

This study was approved by the Animal Ethical and Welfare Committee of Sun Yat-sen University (approval number IACUC-DB-16-0202, Guangzhou, China). Diabetes was induced by intraperitoneal administration of 50 mg/kg STZ (Sigma-Aldrich, St. Louis, MO) dissolved in citrate buffer solution (0.1 mM, pH 4.5) to male Sprague-Dawley (SD) rats weighing 220 g~280 g [28], which were fasted overnight. A week after STZ administration, rats were fasted for 6 hours and blood was obtained by cutting tail tips to measure the glycemic level with a blood glucose meter (Abott). Rats were considered diabetic when fasting blood glucose levels were >14 mmol/L. Diabetic rats were fed with sufficient normal diet and water ad libitum and raised for another 8 weeks before being subjected to surgery.

### 2.2. In Vivo Experiment Protocol

To illustrate HIRI under hyperglycemic settings, normal and diabetic rats of the same weight range (140–150 g) were randomly distributed into 4 groups (*n* = 9 per group): (1) normal sham group; (2) diabetic sham group; (3) normal HIRI group; (4) diabetic HIRI group.

To determine the role of ROS during HIRI in diabetic rats, two classic antioxidants, NAC (300 mg/kg) and NAPDH oxidase inhibitor apocynin (2.5 mg/kg), were dissolved in normal saline and were administered intraperitoneally 1 h before the surgery separately. Confirmed diabetic rats were randomly distributed into 4 groups (*n* = 5 per group): (1) diabetic sham group; (2) diabetic HIRI group; (3) diabetic NAC group; (4) diabetic apocynin group.

### 2.3. Partial Hepatic Ischemia Reperfusion

General anesthesia was induced in all the rats by continuous spontaneous inhalation of 2%~5% volatile anesthetic isoflurane via a mask. After laparotomy, all vessels (hepatic artery, portal vein, and bile duct) to the left and median liver lobes were clamped, according to a previously described method [29]. After 60 min of liver partial ischemia, these vessels were unclamped, and then the abdomen was closed and rats were revived to allow hepatic circulation to be restored for a reperfusion period of 6 hours. Rats were then anesthetized again before being sacrificed by withdrawal of blood from abdominal aorta. The blood collected was centrifuged at 3000 rpm for 10 minutes for serum. The left and median liver lobes were collected for histology and protein extraction described in the following paragraphs.

### 2.4. Histology

Liver paraffin-embedded sections stained were prepared as described previously [30]. Histological severity of HIRI was graded using Suzuki's criteria, in which sinusoidal congestion, hepatocyte necrosis, and ballooning degeneration are grated from 0 to 4. The absence of necrosis, congestion, or centrilobular ballooning is given a score of 0, whereas severe congestion, ballooning degeneration, and >60% lobular necrosis are given a value of 4 [31].

### 2.5. Serum Transferase Activities

The serum alanine transferase (AST) and aspartate transferase (ALT) activities were measured by using the Infinity*™* AST (GOT) Liquid Stable Reagent and Infinity ALT (GPT) Liquid Stable Reagent (Thermo Fisher Scientific Inc.), respectively, according to the manufacturer's instructions. Briefly, 30 *μ*L of serum was mixed with 300 *μ*L of reagent in a 96-well plate and the absorbance at 340 nm was measured with a microplate reader from 1 to 3 minutes. The AST/ALT activity was calculated with the following formula: the activity in U/L = (Δ Absorbance/min) × 1746.

### 2.6. Serum H_2_O_2_ Activities

Serum H_2_O_2_ activities were assessed using KeyGEN H_2_O_2_ Assay Reagent Kit (KGT018, KeyGEN, Nanjing, China) according to manufacturer's instruction. Briefly, 100 *μ*L of sample, distilled water, or standard H_2_O_2_ solution was, respectively, mixed with the reagent provided in the kit in a required order and reacted for 1 minute. Absorbance at 405 nm was measured with a microplate reader and the H_2_O_2_ activity was calculated with the following formula: H_2_O_2_ (mmol/L) = [Abs  (sample) − Abs  (water)]/[Abs  (standard) − Abs  (water)] × 163.

### 2.7. Whole Cell Lysate (WCL)

WCL of liver and hepatocytes were extracted using KeyGEN WCL Assay Kit according to the manufacturer's instruction (KGP2100, KeyGEN, Nanjing, China). Lysis was performed on ice for 15 min, and cell debris was removed by centrifugation at 13,000 ×g at 4°C for 10 min (5810R, Eppendorf AG). The supernatant was recovered as WCL and stored at −80°C.

### 2.8. Hepatic Malondialdehyde (MDA)

Hepatic MDA was assessed with WCL of liver tissues following the instruction of the manufacturer of KeyGEN MDA Assay Reagent Kit (KGT004, KeyGEN, Nanjing, China). Briefly, 200 *μ*L of homogenized liver samples was mixed with the reagent provided in the kit and was processed in boiled water and then ice as stated in the instruction. The sample was then centrifuged and the supernatant was collected for measurement. Absorbance at 532 nm was obtained by a microplate reader.

### 2.9. Immunohistochemistry Staining

Liver paraffin-embedded sections were stained for 8-hydroxydeoxyguanosine (8-OHdG) to evaluate oxidative stress within hepatocytes. The samples were deparaffinized and incubated with monoclonal mouse 8-OHdG Ab (sc-66036, Santa Cruz Biotechnology, Inc.). The integrated optical intensity (IOD) of 8-OHdG (+) within the nuclei of hepatocytes was evaluated by Image-Pro Plus 6.0 (Media Cybernetics, Inc.).

### 2.10. Cell Culture

Normal rat hepatocytes BRL-3A were obtained from the Cell Bank of Shanghai Institutes for Biological Sciences of Chinese Academy of Sciences (category number GNR10) and cultured in low glucose DMEM (Hyclone) containing 10% fetal bovine serum and supplemented with 100 U/mL penicillin and 100 *μ*g/mL streptomycin in a humidified atmosphere in 5% CO_2_ at 37°C. Cells were plated and incubated overnight, before being used in experiment.

### 2.11. Hypoxia/Reoxygenation (H/R)

Cells were seeded and incubated overnight. The medium was replaced by serum-free nonglucose DMEM (Gibco, Thermo Fisher Scientific Inc.). Cellular hypoxic conditions were created and maintained in an airtight incubator (Galaxy 48R, Eppendorf Company) by continuous gas flow with a 95% N_2_ and 5% CO_2_ gas mixture, enabling the percentage of O_2_ to fall to 1% in 15 minutes. After 4 hours of hypoxia, 4 hours of reoxygenation was achieved by continuously flushing with a 95% air and 5% CO_2_ gas mixture and the glucose in the medium was restored.

### 2.12. High Glucose Culture

24 hours after cell plating, the culture media were replaced with a serum-free medium containing 5.5 mM or 25 mM D-glucose. The low glucose culture medium containing 5.5 mM D-glucose was supplemented with 19.5 mM mannitol (Amresco) to adjust the total osmotic pressure to 25 mM. For various purposes, the incubation continued for 6, 24, 48, 72, 96, 120, or 144 hours. As for H/R experiment, cells were incubated in serum-free mediums containing either 5.5 mM or 25 mM D-glucose (the osmotic pressure of which had been balanced as previously stated) for 6 hours before being subjected to hypoxia. At the end of treatment the cells or medium were harvested.

### 2.13. Nuclear Extract Preparation

Cells were washed twice with ice-cold PBS and scraped off from dishes with a rubber cell lifter. Nuclear extracts were prepared according to the instruction of NE-PER Nuclear & Cytoplasmic Extraction Reagent Kit (78833, Thermo Fisher Scientific Inc.). After being extracted, the nuclear and cytoplasmic protein was stored at −80°C.

### 2.14. Cell Viability

Cell viability was assessed by the CCK-8 test (KGA317, KeyGEN, Nanjing, China). Cells were seeded at 1 × 10^5^ into 96-well-plates. After the indicated treatments, 10 *μ*L CCK-8 solution at a 1/10 dilution was added to each well and then the plate was incubated for 2 hours in the incubator. Absorbance at 450 nm was measured with a microplate reader: cell viability (%) = (OD treatment group/OD control group) × 100%.

### 2.15. Cell Cytotoxicity

Cell cytotoxicity was assessed by determining released lactate dehydrogenase (LDH) into the medium by necrotic cells, using Pierce LDH Cytotoxicity Assay Kit (88953, Thermo Fisher Scientific Inc.) according to manufacturer's instruction. Briefly, cultured cells underwent previously stated glucose culture and H/R procedure and subsequently released LDH into the medium after H/R. The medium was transferred to a new plate and mixed with reaction mixture. After 30-minute room temperature incubation, reactions were stopped by adding Stop Solution. Absorbance at 490 nm and 680 nm was measured using a plate-reading spectrophotometer to determine LDH activity. To determine LDH activity, the 680 nm absorbance value (background signal from instrument) was subtracted from the 490 nm absorbance.

### 2.16. ROS

ROS levels were determined using 6-carboxy-2′7′-dichlorodihydrofluorescein diacetate (DCFH-DA) (D6883, Sigma-Aldrich, St. Louis, MO). Cells were washed with ice-cold PBS and incubated with 10 *μ*M DCFH-DA for 30 min. Then, the medium was discarded and cells were washed with ice-cold PBS in the dark, and ROS generation was evaluated by the fluorescence intensity measured by fluorescence spectrometry (SpectraMax M5, Molecular Devices, USA). The excitation wavelength was 504 nm and emission wavelength was 529 nm.

### 2.17. Enzyme-Linked Immunosorbent Assay (ELISA)

Rat-specific ELISA kits were applied to determine hepatic and medium tumor necrosis factor *α* (TNF-*α*, SEA133Ra, USCN, Cloud-Clone Corp., Wuhan, China), monocyte chemokine protein-1 (MCP-1, SEA087Mi, USCN, Cloud-Clone Corp., Wuhan, China), and hepatic interleukin-6 (IL-6, SEA079Ra, USCN, Cloud-Clone Corp., Wuhan, China) and levels according to the manufacturer's instructions. Absorbance all measured at 450 nm. Hepatic 8-isoprostane level was determined using Cayman: 8-Isoprostane EIA Kit (number 516351, Cayman Chemical, USA) according to the manufacturer's instruction. The absorbance was measured at a wave length of 420 nm.

### 2.18. Western Blotting Assays

Western blotting assays were performed as described before [32]. Briefly, liver or hepatocyte WCL or nucleus extract were collected and the protein concentration was measured by Bradford assay. The samples were subjected to electrophoresis and transformation to polyvinylidene fluoride membranes. These membranes were blocked in 5% milk for 1 h, incubated with different primary antibodies overnight at 4°C, and then rinsed and incubated with secondary horseradish peroxidase-conjugated antibody for 1 h at room temperature. Antigen antibody complexes were then visualized using ECL kit (35050, Thermo Scientific Inc.).

The primary antibodies used here include those against nuclear factor-erythroid 2-related factor 2 (Nrf2, 1 : 1000, Abcam, Cambridge, MA), nuclear factor of kappa light polypeptide gene enhancer in B-cells (NF-*κ*B, 1 : 1000, Cell Signaling Technology (CST), Danvers, MA), total NF-*κ*B inhibitor, alpha (I*κ*B*α*, 1 : 2000, CST), phosphor-I*κ*B*α* (1 : 500, CST), heme oxygenase-1 (HO-1, 1 : 250, Santa Cruz), and NAD(P)H:quinone oxidoreductase 1 (NQO1, 1 : 250, Santa Cruz).

### 2.19. Statistical Analysis

Results were processed by SPSS 13.0 (SPSS Inc.). Measurable data are expressed as means ± standard error of the mean (SEM). Pathological scores are expressed as median with interquartile range. Statistical analyses of measurable data were performed with the independent *t*-test, and we performed Mann-Whitney *U* test to analyze pathological scores. A *P* value less than 0.05 was considered significant.

## 3. Results

### 3.1. Hyperglycemia Aggravates HIRI in Diabetic Rats

**Figure 1:**
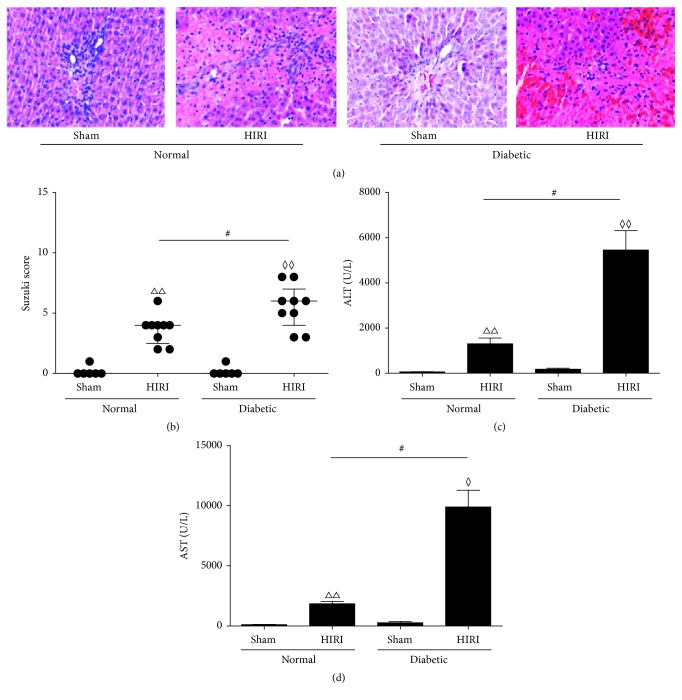
HIRI after 60 minutes of ischemia followed by 6 hours of reperfusion in control or diabetic rats. ((a), (b)) Hepatic pathological sections (200x) and pathology score by Suzuki's criteria. ((c), (d)) Serum ALT and AST levels. Measurable data are expressed as mean ± SEM (*n* = 9 per group). Pathology scores are expressed as medium with interquartile range. ^◊^ *P* < 0.05 versus diabetic sham group; ^◊◊^ *P* < 0.01 versus diabetic sham group; ^#^ *P* < 0.05 versus normal HIRI group. HIRI: hepatic ischemia reperfusion injury; ALT: alanine aminotransferase; AST: aspartate aminotransferase; sham: sham operating group.

 Behrends et al. have proved that acute hyperglycemia induced by single intraperitoneal injection of glucose could worsen HIRI [33]. To establish a more stable hyperglycemia model, we adopted STZ-induced type 1 diabetes model to intensively study the effect of glucose overload. We measured the glycemic level of normal and diabetic rats subjected to sham or partial hepatic I/R, respectively, and confirmed consistent significant hyperglycemia in STZ-induced diabetic rats compared to normal rats (Table 1). There were no significant differences with regard to hepatic histological findings between diabetic sham group and normal sham group (Figures 1(a) and 1(b)). However, when subjected to IR, more severe hepatocyte necrosis, sinus congestion, and hepatocyte ballooning were observed in diabetic group (Figures 1(a) and 1(b), *P* < 0.05). The trend of serum ALT and AST was consistent with histological findings (Figures 1(c) and 1(d), *P* < 0.05), which also indicated that hyperglycemia resulted in more serious HIRI.

### 3.2. Hyperglycemia Increases Baseline Lipid Peroxidation and Inflammatory Cytokines in Diabetic Rats and Further Aggravated Them after HIRI

The unifying hypothesis of diabetic complication places ROS at the center of the deleterious effect of hyperglycemia [13]. Liver is particularly prone to oxidative/nitrosative stress due to its high metabolic rate and because hepatocytes are rich in ROS/RNS-producing mitochondria, cytochrome P450 (CYP) enzymes, and inducible nitric oxide synthase (iNOS). Increased oxidative/nitrosative stress has been a key contributor of hepatopathology relevant to diabetes such as NAFLD, viral hepatitis, and alcoholic fatty liver disease [34]. So we found it necessary to determine the extent of oxidative stress between diabetic and normal rats.

**Figure 2:**
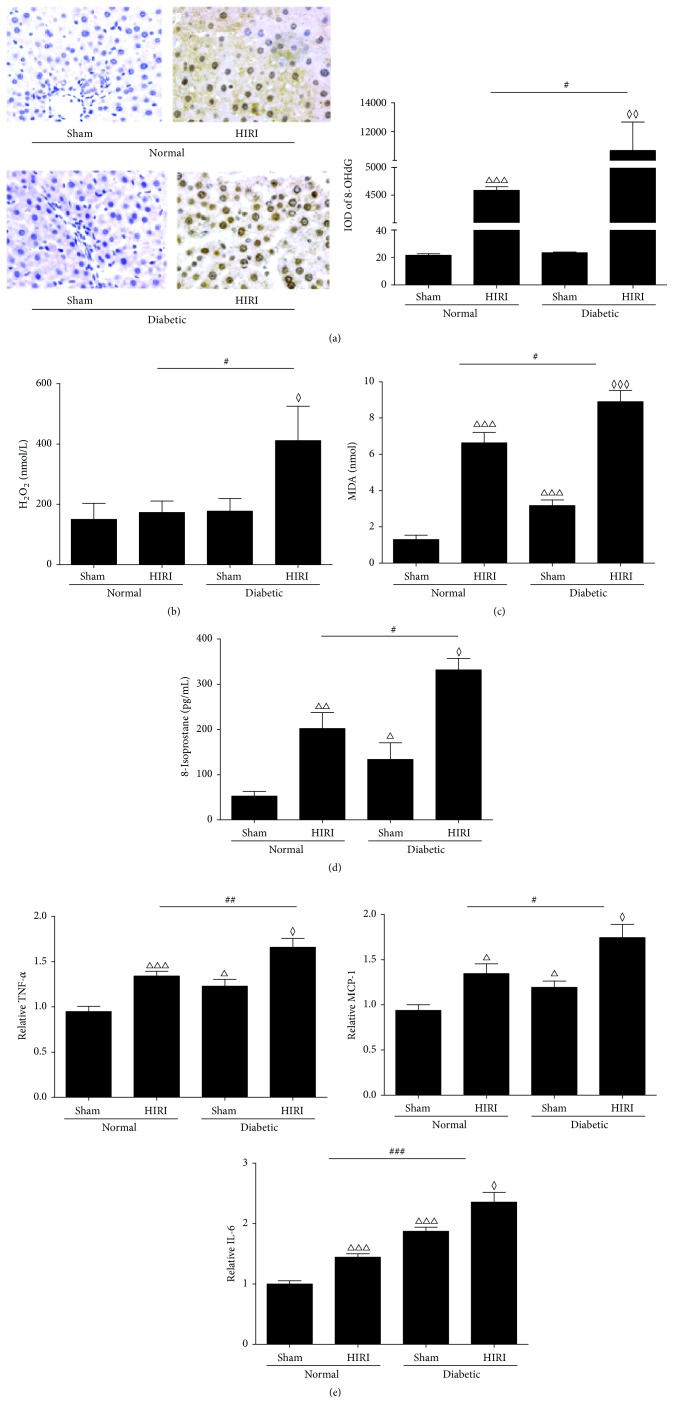
Hepatic oxidative stress and inflammation in normal or diabetic rats. (a) Nuclear 8-OHdG (400x) detected by IHC staining and IOD assessed by Image-J. (b) Serum H_2_O_2_ levels. (c) Hepatic MDA levels. (d) Hepatic 8-isoprostane determined by ELISA. (e) Hepatic inflammatory cytokines determined by ELISA. Data are expressed as mean ± SEM (*n* = 9 per group). ^△^ *P* < 0.05 versus normal sham group; ^△△^ *P* < 0.01 versus normal sham group; ^△△△^ *P* < 0.001 versus normal sham group; ^◊^ *P* < 0.05 versus diabetic sham group; ^◊◊^ *P* < 0.01 versus diabetic sham group; ^◊◊◊^ *P* < 0.001 versus diabetic sham group; ^#^ *P* < 0.05 versus normal HIRI group; ^##^ *P* < 0.01 versus normal HIRI group; ^###^ *P* < 0.001 versus normal HIRI group. HIRI: hepatic ischemia reperfusion injury; 8-OHdG: 8-hydroxydeoxyguanosine; IOD: integrated optical intensity; IHC: immunohistochemistry; H_2_O_2_: hydrogen peroxide; MDA: malondialdehyde; TNF-*α*: tumor necrosis factor *α*; MCP-1: monocyte chemokine protein-1; IL-6: interleukin-6; ELISA: enzyme-linked immunosorbent assay; sham: sham operating group.

As shown in Figure 2, serum H_2_O_2_ and nuclear 8-OHdG were higher in diabetic HIRI group compared to normal HIRI group (Figures 2(a) and 2(b), *P* < 0.05) which implicated excessive ROS generation and consequent aggravated DNA injury in diabetic rats. The baseline level of lipid peroxidation product, hepatic 8-isoprostane and MDA, increased significantly in diabetic rats compared to normal rats and soared after IR (Figures 2(c) and 2(d), *P* < 0.05).

Subclinical inflammation has also been reported among the basic changes within diabetic patients [35]. We determined several common inflammatory cytokines including MCP-1, IL-6, and TNF-*α* in hepatic WCL, and it turned out they were also significantly higher in diabetic rats and further increased after IR (Figure 2(e), *P* < 0.05).

### 3.3. High Glucose Culture before H/R Increased the Vulnerability of BRL-3A Hepatocytes to H/R Injury

**Figure 3:**
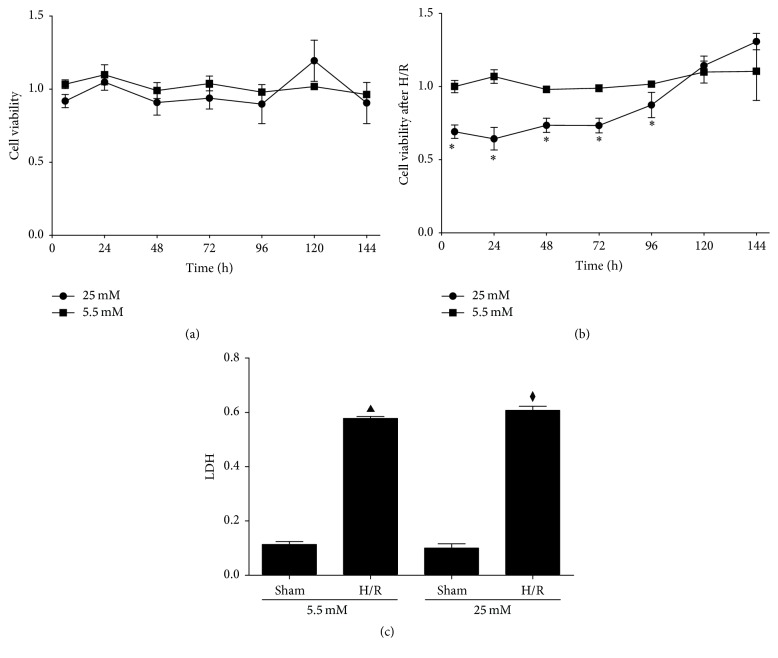
High glucose culture exerted deleterious effect upon BRL-3A hepatocytes after hypoxia/reoxygenation. (a) Cell viability under normoxic circumstances after being treated with serum-free medium containing 5.5 Mm and 25 mM D-glucose, respectively. (b) Cell viability under redox circumstances after being treated with serum-free medium containing 5.5 mM and 25 mM, respectively. (c) LDH levels in the medium after hypoxia/reoxygenation. ^*∗*^ *P* < 0.05 versus 5.5 mM D-glucose group; ^▲^ *P* < 0.05 versus 5.5 mM control group; ^⧫^ *P* < 0.05 versus 25 mM control group. H/R: hypoxia/reoxygenation; LDH: lactate dehydrogenase.

We subjected BRL-3A hepatocytes to 5.5 mM and 25 mM of D-glucose culture for 6–96 hours, and it turned out that the cell viability of BRL-3A was similar between 2 groups in normoxic environment (Figure 3(a)). However, after H/R, the viability of BRL-3A hepatocytes was significantly hampered after 6, 24, 48, 72, and 96 hours of 25 mM glucose pretreatment (Figure 3(b), *P* < 0.05). H/R could comparably increase the LDH leakage in both groups (Figure 3(c)).

### 3.4. High Glucose Culture Increased ROS and Cytokines under Normoxic Settings and Further Elevated Them after H/R

**Figure 4:**
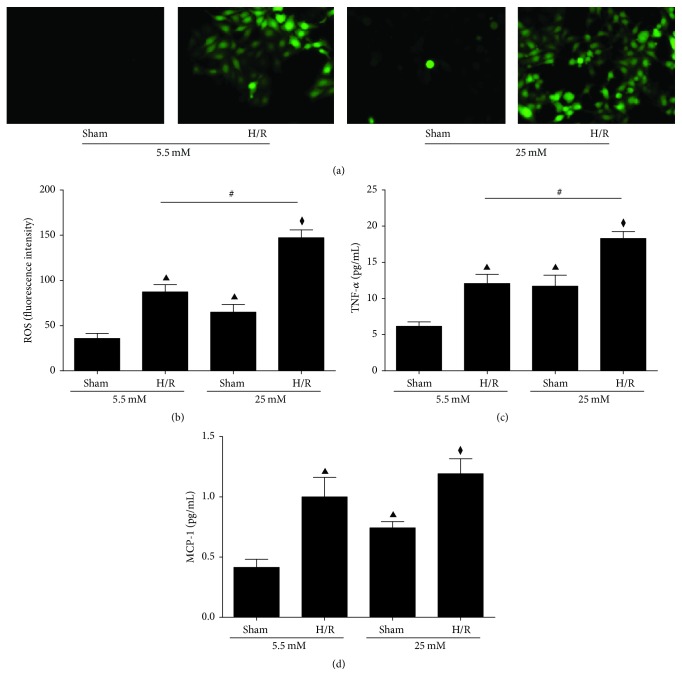
High glucose culture increases ROS generation and cytokines release within BRL-3A hepatocytes. ((a), (b)) Intracellular ROS stained by 6-carboxy-2′7′-DCFH-DA (200x) and the fluorescence intensity was detected by fluorescence spectrophotometer. ((c), (d)) TNF-*α* and MCP-1 in the culture medium. ^▲^ *P* < 0.05 versus 5.5 mM control group; ^⧫^ *P* < 0.05 versus 25 mM control group; ^#^ *P* < 0.05 versus 5.5 mM H/R group. H/R: hypoxia/reoxygenation; ROS: reactive oxygen species; TNF-*α*: tumor necrosis factor *α*; MCP-1: monocyte chemokine protein-1.

It has been proposed that excessive glucose induces ROS in endothelial cells mainly by activation of protein kinase C isoforms, increased formation of glucose-derived advanced glycation end-products (AGEs), and increased glucose flux through the aldose reductase pathway or polyol pathways [36, 37]. Here we demonstrated in hepatocytes that ROS generation was also increased after 25 mM glucose culture. H/R stress resulted in excessive production of ROS in both 5.5 mM and 25 mM glucose pretreated group. However, the fluorescent intensity of ROS was significantly higher and further enhanced after H/R in 25 mM group compared to 5.5 mM group (Figures 4(a) and 4(b), *P* < 0.05). This implicates that the stress of acute H/R can amplify existing oxidative stress induced by excessive glucose, therefore causing more severe HIRI compared to control.

As has been observed in in vivo HIRI models, inflammatory cytokines, including MCP-1 and TNF-*α* in culture medium, were both significantly increased after exposure to 25 mM D-glucose under normoxic settings as well (Figures 4(c) and 4(d), *P* < 0.05). After H/R, in 25 mM pretreated cells, TNF-*α* was significantly higher compared to 5.5 mM group (Figure 4(c), *P* < 0.05).

### 3.5. High Glucose Culture Triggered NF-*κ*B Translocation within Normoxic and Hypoxic-Redox BRL-3A Cells

**Figure 5:**
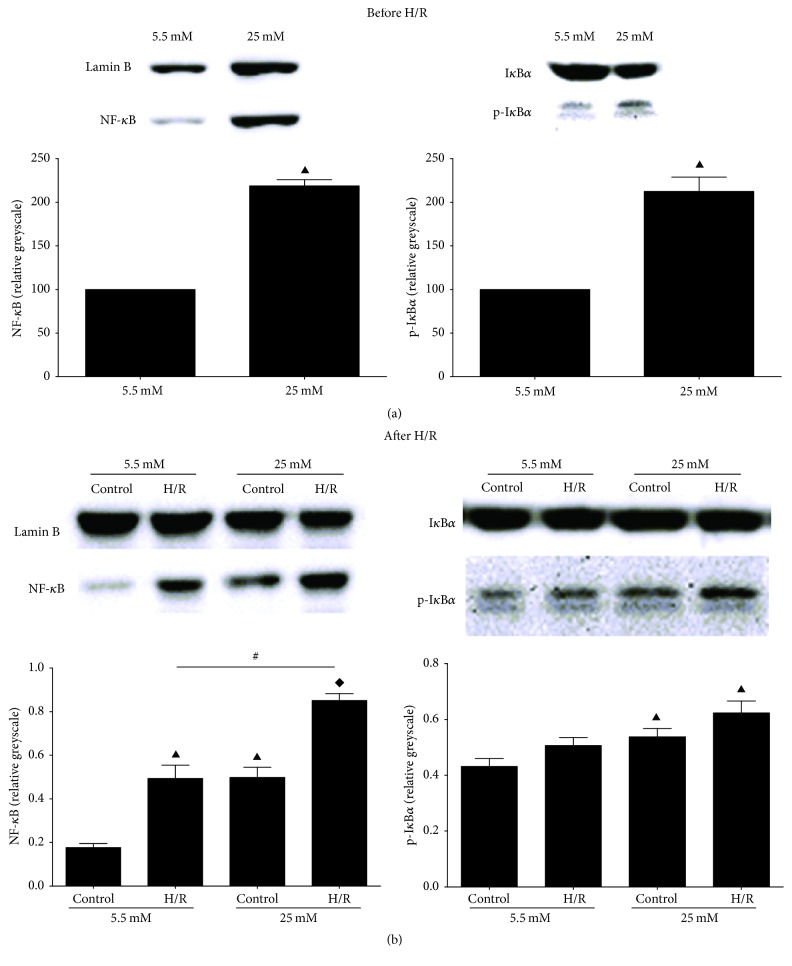
NF-*κ*B signaling related protein alterations determined by western blotting before and after H/R. (a) NF-*κ*B and its cytoplasm inhibitor I*κ*B*α* after 6 hours of 5.5 mM or 25 mM D-glucose culture before H/R. (b) NF-*κ*B and I*κ*B*α* after H/R. ^▲^ *P* < 0.05 versus 5.5 mM control group; ^*◆*^ *P* < 0.05 versus 25 mM control group; ^#^ *P* < 0.05 versus 5.5 mM H/R group. H/R: hypoxia/reoxygenation; NF-*κ*B: nuclear factor of kappa light polypeptide gene enhancer in B-cells; I*κ*B*α*: total inhibitor alpha; p-I*κ*B*α*: phosphor-I*κ*B*α*.

Besides direct damage to cell components, ROS are also pivotal signals in various cellular pathways including NF-*κ*B pathway [38]. NF-*κ*B is an important transcription factor that reacts to redox signals and regulates the expression of many inflammatory cytokines including MCP-1, IL-6, and TNF-*α* [39, 40]. Compared with 5.5 mM group, I*κ*B*α* phosphorylation and nuclear NF-*κ*B were higher before H/R after 6 hours of 25 mM D-glucose culture (Figure 5(a), *P* < 0.05), which explained the consistently increased cytokines in medium. After H/R, the nuclear NF-*κ*B in 25 mM H/R group was significantly increased compared to 5.5 mM H/R group (Figure 5(b), *P* < 0.05). Thus, high glucose culture before H/R not only could accelerate NF-*κ*B activation but also increased the translocation of NF-*κ*B after H/R. The subsequently upregulated proinflammatory cytokines could activate neutrophils and Kupffer cells to trigger profound cytotoxic immune response [41].

### 3.6. High Glucose Culture Activated Nrf2 Translocation within Normoxic and Hypoxic-Redox BRL-3A Cells without Affecting the Expression of NQO1 and HO-1

**Figure 6:**
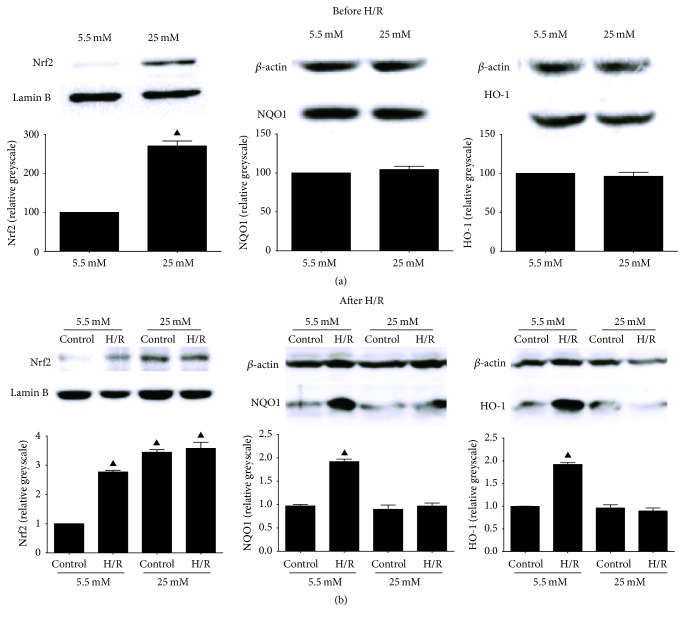
Nrf2 signaling related protein alterations determined by western blotting before and after HR. (a) Nrf2 and its downstream antioxidases NQO1 and HO-1 after 6 hours of 5.5 mM or 25 mM D-glucose culture before H/R. (b) Nrf2, NQO1, and HO-1 after H/R. ^▲^ *P* < 0.05 versus 5.5 mM control group. H/R: hypoxia/reoxygenation; Nrf2: nuclear factor-erythroid 2-related factor 2; HO-1: heme oxygenase-1; NQO1: NAD(P)H:quinone oxidoreductase 1.

Nrf2 is another redox-sensitive transcription factor that mainly regulates the expression of antioxidases including HO-1 and NQO1 to counter harmful stimuli including ROS [42]. Here we demonstrated in BRL-3A hepatocytes that nuclear Nrf2 was significantly higher before H/R after 6 hours of 25 mM D-glucose culture compared with 5.5 mM group (Figure 6(a), *P* < 0.05). Nevertheless, as downstream oxidase of Nrf2, the expression of NQO1 and HO-1 was comparable between low and high glucose pretreated groups. Consistently, while NQO1 and HO-1 were upregulated after H/R in 5.5 mM H/R group compared to normoxic control, they remained unaffected in 25 mM H/R group (Figure 6(b)).

### 3.7. NAC and Apocynin Pretreatment Ameliorated HIRI and Oxidative Stress and Inflammation

With these findings, we postulate that ROS lies in the center of the amplified inflammation cascades and aggravated HIRI. ROS scavengers including NAC and apocynin have been suggested to be a solution to ameliorate or reverse the deleterious effect of overly triggered oxidative stress [43, 44]. N-Acetyl-cysteine serves as a prodrug to L-cysteine, which is a precursor to the biologic antioxidant glutathione. Hence, administration of NAC replenishes reduced glutathione (GSH) [45]. The GSH synthesis is controlled by the activity of *γ*-glutamyl cysteine ligase (*γ*-GCL) and by the availability of cysteine. *γ*-GCL is composed of two subunits: the glutamate-cysteine ligase complex modifier subunit (GCLM) and the GCL catalytic subunit (GCLC). Nrf2 regulates the expression of these subunits [42]. Apocynin is an inhibitor of NADPH oxidase activity and thus is effective in preventing reduction of O_2_ to superoxide (O_2_^−•^), in human neutrophilic granulocytes. We intended to examine whether HIRI in diabetic rats could be ameliorated by either partial restoration of hampered Nrf2 pathway or suppression of ROS generation.

**Figure 7:**
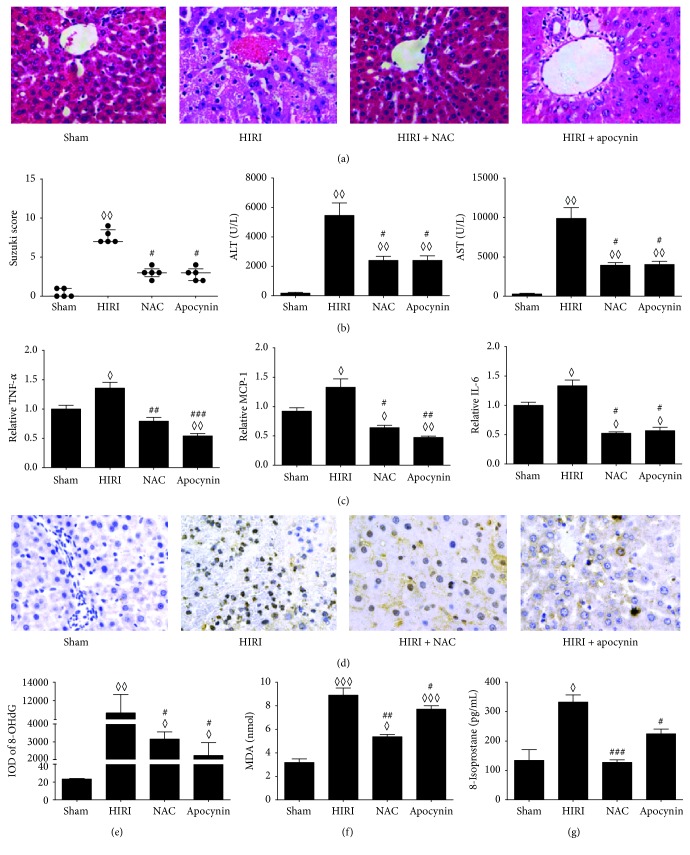
Hepatic ischemia reperfusion injury, oxidative stress, and inflammation in NAC or apocynin pretreated diabetic rats. ((a), (b)) Hepatic pathology sections (200x), pathological scores, and serum transferase. (c) Hepatic inflammatory cytokines levels. ((d), (e)) Nuclear 8-OHdG (400x) detected by IHC staining and IOD assessed by Image-J; (f) MDA levels; (g) 8-isoprostane levels by ELISA. Measurable data are expressed as mean ± SEM (*n* = 5 per group). Pathology scores are expressed as medium with interquartile range. ^◊^ *P* < 0.05 versus sham group; ^◊◊^ *P* < 0.01 versus sham group; ^◊◊◊^ *P* < 0.001 versus sham group; ^#^ *P* < 0.05 versus HIRI group; ^##^ *P* < 0.01 versus HIRI group; ^###^ *P* < 0.001 versus HIRI group. HIRI: hepatic ischemia reperfusion injury; NAC: N-acetyl-L-cysteine; IHC: immunohistochemistry; 8-OHdG: 8-hydroxydeoxyguanosine; IOD: integrated optical intensity; MDA: malondialdehyde; TNF-*α*: tumor necrosis factor *α*; MCP-1: monocyte chemokine protein-1; IL-6: interleukin-6; ELISA: enzyme-linked immunosorbent assay; sham: sham operating group.

In the present study, we found out that NAC and apocynin administered intraperitoneally before IR could both ameliorate HIRI in diabetic rats, indicated by lower pathological scores and lower serum ALT and AST (Figures 7(a) and 7(b), *P* < 0.05). NAC and apocynin pretreatment could also lower the expressions of hepatic IL-6, MCP-1, and TNF-*α* (Figure 7(c), *P* < 0.05) compared with HIRI group. The nuclear 8-OHdG significantly decreased in NAC and apocynin pretreated group compared with HIRI group (Figures 7(d) and 7(e), *P* < 0.05). The same went for hepatic 8-isoprostane and MDA (Figures 7(f) and 7(g), *P* < 0.05), which indicated a decrease in lipid peroxidation.

## 4. Discussion

Hyperglycemia alone under normoxic condition does not significantly impact the viability of hepatocytes. Chen et al. demonstrated that Chang liver cells in mannitol-balanced 5.5 mM, 25 mM, and 100 mM glucose media after at least 3 weeks showed no significant variation in viability and apoptosis among the three culture conditions [21]. However, hyperglycemia is associated with chronic oxidative stress [13]. In the cardiac tissues of both type 1 and type 2 diabetes, an elevated baseline of ROS induced by hyperglycemia was identified [46]. Glucose induce ROS mainly by activation of protein kinase C isoforms, increased formation of glucose-derived advanced glycation end-products (AGEs), and increased glucose flux through the aldose reductase pathway or polyol pathways [36, 37]. Consistently, the present study demonstrated that both in vivo and in vitro high glucose exposure could increase baseline hepatic oxidative stress.

Chronic oxidative stress may promote the onset or progression of chronic liver diseases which if not treated properly are likely to advance to end-stage liver diseases requiring surgical intervention [20, 21]. However, little is known about the impact of chronic oxidative stress caused by hyperglycemia on HIRI. During reperfusion, enormous ROS are generated due to sudden restoration of oxygen and exert direct deleterious effects on cells through lipid peroxidation, protein degradation, and DNA damage [1]. The present study demonstrated that hepatic oxidative stress was further exacerbated after HIRI or H/R in both in vivo and in vitro high glucose settings. In line with soaring ROS generation, hepatocytes in high glucose environment predisposed to HIRI or H/R injury demonstrated by higher pathological score and serum transferase or lower cell viability. This implicates that the stress of acute IR can amplify existing oxidative stress induced by glucose overloads, therefore causing more severe HIRI compared to control.

In addition to direct damage to cell components, ROS can also activate various cellular pathways including NF-*κ*B pathway [38]. The inactive NF-*κ*B is sequestered in the cytoplasm with an inhibitor protein, I*κ*B. In a conventional activation pathway, I*κ*B is phosphorylated by I*κ*B kinases (IKKs) in response to different activators, including ROS, and subsequently degraded thus liberating the active NF-*κ*B complex to nucleus [39]. We observed phosphorylated I*κ*B*α*, increased NF-*κ*B translocation into nucleus after high glucose culture before H/R, which explained the consistently increased cytokines in medium. The result of in vitro study was similar to that of in vivo study where hepatic cytokines increased at baseline and strikingly rose after IR in diabetic rats, suggesting high glucose induced activation of NF-*κ*B might also be involved in HIRI in diabetic rats. Clinically NF-*κ*B related cytokines, TNF-*α*, IL-6, and MCP-1, were found increased in patients with type 2 diabetes [35]. These proinflammatory cytokines could activate neutrophils and Kupffer cells to trigger profound cytotoxic immune response [41].

Nrf2 is another redox-sensitive transcription factor that mainly regulates the expression of antioxidases including HO-1 and NQO1 to counter harmful stimuli including ROS [42]. It has been reported that Nrf2 pathway activates in high glucose stimulated cardiomyocytes endothelial cells and renal mesenchymal cells as intrinsic defense against oxidative stress [42, 47, 48]. However, in our present study, high glucose could increase the Nrf2 translocation in hepatocytes but did not alter the expression of HO-1 and NQO1. Moreover, in contrast to its further increase and consequent upregulation of HO-1 and NQO1 after H/R in low glucose pretreated hepatocytes, nuclear Nrf2 in high glucose pretreated hepatocytes was not significantly increased following H/R and neither were its downstream antioxidases. The results implicate that the antioxidative ability of Nrf2 pathway might be hampered after high glucose exposure.

Recent evidence indicates that NF-*κ*B may directly repress Nrf2 signaling at the transcription level [49, 50]. DNase I footprint assays using purified transcription factors revealed the presence of NF-*κ*B and AP-2 binding sites in the proximal part of the promoter region of the human HO-1 gene [51]. Therefore, it is reasonable to postulate that ROS overproduction by high glucose could initiate both NF-*κ*B and Nrf2 activation, while the former prevailed and frustrated the function of Nrf2. However, the exact mechanisms of NF-*κ*B and Nrf2 interaction under hyperglycemic settings remain to be further elucidated.

With these findings, we postulate that ROS lies in the center of the amplified inflammation cascades and aggravated HIRI. ROS scavengers including NAC and apocynin have been suggested to be a solution to ameliorate or reverse the deleterious effect of overly triggered oxidative stress [43, 44]. NAC and apocynin administered intraperitoneally before IR could ameliorate hepatic injury in diabetic rats along with decreased inflammatory cytokines, attenuated lipid peroxidation, and ROS-inflicted DNA injury compared with the HIRI controls. The present results are consistent with those of other researchers which reported the long-term oral administration of NAC and Allopurinol showed synergistic protective effect on diabetic ischemic cardiac tissues [52]. It is therefore a promising strategy to alleviate HIRI by countering preoperative chronic oxidative stress in diabetes before IR.

One of the limitations of this study may lie in the choice of animal models used to represent chronic hyperglycemia. STZ-induced diabetes is a model of type 1 diabetes mellitus, within which the cause of hyperglycemia is attributed to insulin insufficiency due to STZ toxicity upon islet *β*-cells [53]. The dosage, approach, and frequency of STZ administration vary in a broad range [54]. In the current study, we chose to give a single dose of 50 mg/kg of STZ administered intraperitoneally, which is a considerably mild dose compared to those used in other studies. We provided sufficient normal diet and water for the confirmed diabetic rats and measured the fasting, random, and preoperative glycemic levels to prevent hypoglycemia. Since persistent hyperglycemia is a major manifestation of all kinds of diabetes, the primary aim of our study was to examine the impact of glucose excess on HIRI.

Another issue that might be of concern is the potential protection of insulin in hyperglycemia induced aggravation of HIRI. Insulin is undoubtedly the first-line treatment for preoperative and intraoperative hyperglycemia. Preoperative insulin treatment is definitely effective in compensating *β*-cell function and lowering blood glucose in STZ-induced type 1 diabetic models. Rocha et al. demonstrated that insulin led to the significant reduction of the serum concentration of AST, ALT, gamma-glutamyl transferase (GGT), and LDH in rat HIRI model. Nevertheless, these beneficial effects were found to be independent from blood glucose levels and may have been attributed to inhibition of GSK-3*β* [55]. Our focus of the current study, however, was mainly on the role of hyperglycemia, as an initial trigger of chronic oxidative stress, which will lead to further exacerbation of I/R injury. However, persistent hyperglycemia is very common when surgical stress is imposed upon diabetic patients, sometimes even in normal patients, since the secretion of catecholamine would aggravate insulin resistance, thus reducing the efficacy of exogenous insulin infusion. Moreover, the attempt to lower blood glucose might not be enough or in time to reverse the deleterious effects, for instance, the formation of excessive ROS and amplified inflammation, which are already imposed on the rendered liver. Therefore, we intended to find a solution to ameliorate the consequence caused by high glucose as it may be more practical in the context of surgery. Nevertheless, we cannot deny the potential protection offered by insulin during the preoperative preparation, which has proven to be very meaningful for the timing of elective surgery on diabetic patients. We will conduct more researches to study the effect of insulin-related pathways on diabetic HIRI in the future.

## 5. Conclusion

**Figure 8:**
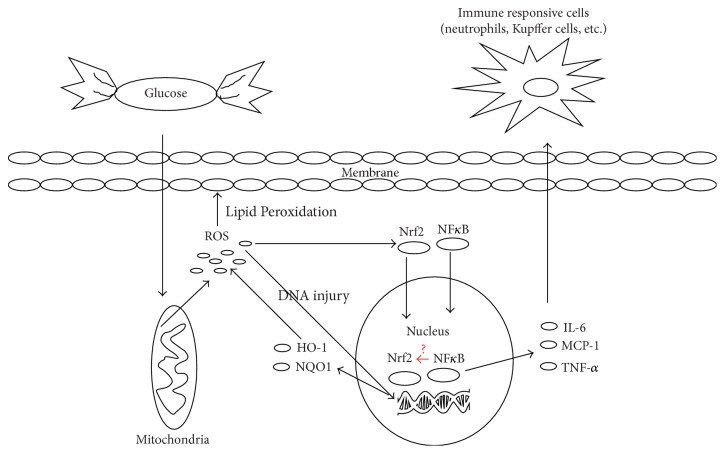
The proposed mechanisms of hyperglycemia induced aggravation of HIRI. Chronic oxidative stress represented by generation of ROS was induced by excessive glucose. The amplified inflammation may be attributed to NF-*κ*B translocation most probably induced by chronic oxidative stress. Hampered antioxidative ability of Nrf2 pathway may also be involved in aggravated HIRI after high glucose exposure. The potential inhibitory interaction between NF-*κ*B and Nrf2 at transcription level may be a possible explanation.

In summary, we demonstrated that hyperglycemia could aggravate HIRI, and the exacerbation was related to chronic oxidative stress and inflammation induced by excessive glucose (Figure 8). The amplified inflammation may be attributed to NF-*κ*B translocation most probably induced by chronic oxidative stress. Hampered antioxidative ability of Nrf2 pathway may also be involved in aggravated HIRI after high glucose exposure. Inhibitory interaction between NF-*κ*B and Nrf2 at transcription level may be a possible explanation, though the exact mechanisms of the interaction of these two transcription factors under hyperglycemic settings remain to be studied. Antioxidant precondition may be a potential therapy to alleviate diabetic HIRI.

## Figures and Tables

**Table 1:** Fasting, random, and preoperative glycemic level of normal and diabetic rats. ^△△△^ *P* < 0.001 versus normal sham group; ^###^ *P* < 0.001 versus normal HIRI group. All glycemic measurement was obtained from blood dripped from cut tail tip and determined by a blood glucose meter (Abott). Fasting glucose was measured when rats were fasted for 6 hours. Random glucose was measured without deliberate interruption of food and water supply. Preoperative glucose was measured after the rats were anesthetized and before laparotomy.

Glycemia (mmol/L)	Normal		Diabetic	
	Sham (*n* = 9)	HIRI (*n* = 9)	Sham (*n* = 9)	HIRI (*n* = 9)
Fasting	4.53 ± 0.35	4.52 ± 0.34	14.48 ± 0.61^△△△^	14.55 ± 0.56^###^
Random	7.20 ± 0.57	8.62 ± 0.68	23.60 ± 2.29^△△△^	22.55 ± 1.89^###^
Preoperative	4.40 ± 0.38	4.43 ± 0.36	14.43 ± 0.62^△△△^	14.40 ± 0.48^###^
